# Extreme longevity revealed by 24-year genetic mark–recapture challenges conservation of endangered sea cucumbers

**DOI:** 10.1016/j.isci.2026.117505

**Published:** 2026-09-15

**Authors:** Sven Uthicke, Maria Gomez Cabrera, Maria Byrne, Steven W. Purcell

**Affiliations:** 1 Australian Institute of Marine Science, PMB No 3, Townsville, QLD 4810, Australia; 2 Marine Invertebrate Futures Group, School of Life and Environmental Sciences, The University of Sydney, Sydney, NSW 2006, Australia; 3 National Marine Science Centre, Southern Cross University, Coffs Harbour, NSW 2450, Australia

**Keywords:** longevity, invertebrate overfishing, conservation

## Abstract

Coral reef sea cucumbers are heavily exploited despite the precarious conservation status of many species, yet limited life-history knowledge constrains effective management. We used genetic mark–recapture to estimate longevity, mortality, and growth in the endangered, CITES-listed black teatfish *Holothuria* (*Microthele*) *whitmaei* by resampling a population after 24 years. Most individuals (60%) in 2024 were genetically identical to those sampled in 2000, indicating extremely low adult mortality (∼0.04 per year), a conservative mean lifespan of ∼24–27 years, and longevity exceeding 100 years. Most recaptured individuals showed negligible or negative growth. Current harvest models for tropical sea cucumbers do not account for such extreme longevity and likely overestimate sustainable yields. Our results suggest that exploitation of long-lived reef invertebrates is unsustainable under prevailing harvest rates and environmental stress, highlighting the need to reassess productivity-based fisheries frameworks more broadly.

## Highlights

• Genetic matching demonstrated 60% of black teatfish adults survived after 24 years
• Most individuals showed negative growth—shrinking in body weight and length
• Longevity exceeded 100 years, and mortality was exceptionally low (*M* = 0.04/year)
• Extreme longevity challenges fishery harvest models and conservation planning

## Graphical abstract

**Figure:**
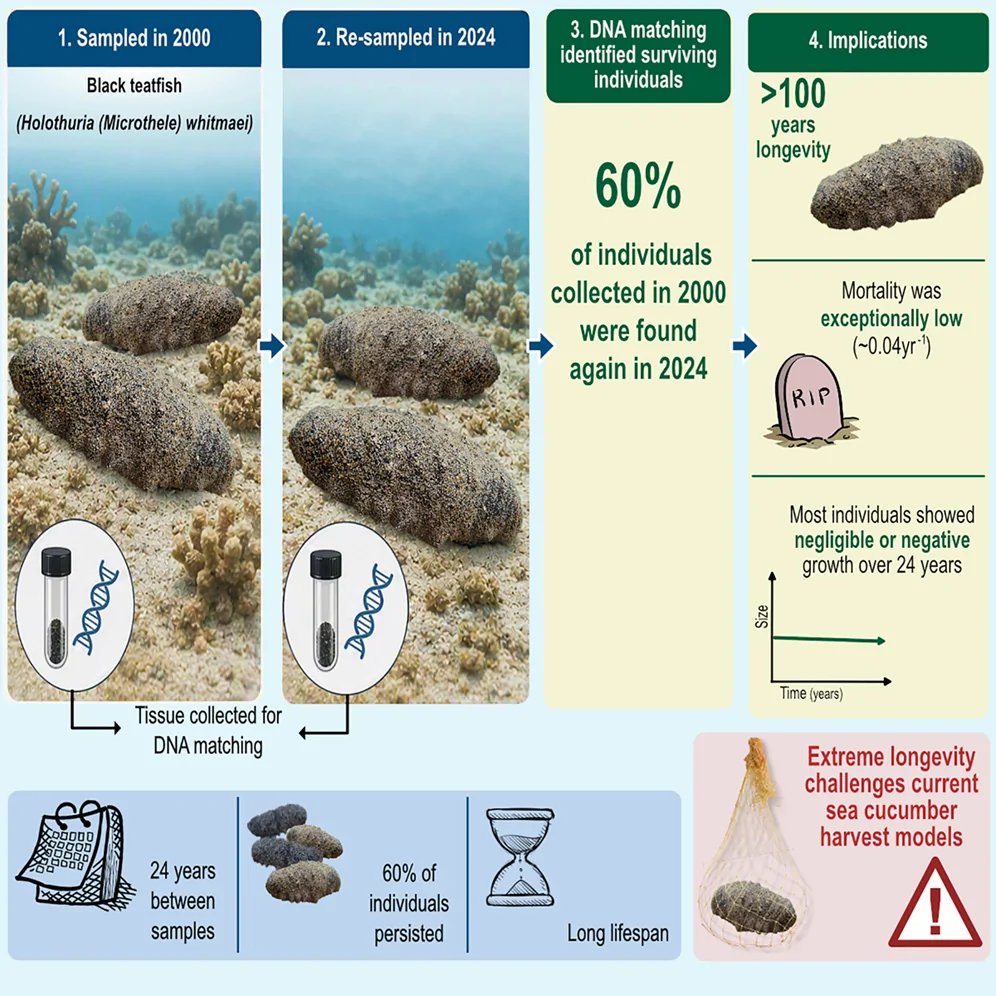

## Teaser

Marine organism; Nature conservation; Zoology

## Introduction

Long lifespans, often exceeding those of humans, are known from fishes such as the Greenland shark and Pacific rockfishes, yet the oldest living animals in the sea are invertebrates.^1^ Some classes of invertebrates show little sign of senescence, possessing resistance to injury and disease that allows some species to exhibit extreme lifespans.^2^^,^^3^ Black corals, glass sponges, ocean quahog clams, and deep-sea tube worms can live more than 300 years. Certain echinoderms can live for many decades or more. Data on lifespans inform estimates of generation length, which is integral for classifying extinction risks, and inform management and conservation plans.^4^^,^^5^

Definitive maximum-age studies are lacking for most echinoderm taxa, many of which are commercially harvested. Sea urchins have been found to grow indeterminately, and their telomeres do not shorten with age.^6^^,^^7^ In addition, several sea stars and sea cucumbers reproduce asexually though autonomy or transverse fission and regeneration, practically extending the lifespan of a genotype indefinitely.^8^^,^^9^^,^^10^ The regenerative capacity of certain echinoderms to re-grow lost or damaged appendages is well known.^11^ For echinoderms, substantial longevity has mostly been reported for temperate species. Using radiocarbon dating, the sea urchin *Mesocentrotus franciscanus* from the northeast Pacific was found to live for well over 100 years.^12^ In the northern Barents Sea, the sea urchin, *Strongylocentrotus pallidus*, possess a lifespan exceeding 40 years,^13^ and the Arctic brittle star, *Ophiopleura borealis*, also lives multiple decades.^14^ Lifespans of tropical echinoderms, including those that occur on coral reefs, have rarely been studied.

Sea cucumbers have key ecological functions in many marine ecosystems, with tropical species playing important roles in coral reef health.^15^^,^^16^ Yet many of these animals are heavily overexploited in the bêche-de-mer trade for luxury Asian seafood markets, leading to seven species being listed on CITES Appendix II and classified as vulnerable or endangered on the IUCN Red List.^17^ Overfishing of coral reef bêche-de-mer sea cucumbers is likely to add to reef degradation due to cumulative pressures dominated by climate change.^18^

In addition to longevity, natural instantaneous mortality (*M*) is the most important parameter for fisheries stock assessment and conservation measures but is very difficult to estimate. In general, instantaneous mortality can increase with age (often increasing after maturity), as in most mammals, including humans.^19^^,^^20^^,^^21^^,^^22^ In sea urchins, cases with declining mortality over time (“negative senescence”) have also been documented.^23^ However, most fecund marine organisms, including fishes and invertebrates, suffer high mortality rates in early life (larvae and juveniles), followed by constant mortality rates after adulthood.^24^ Age-specific mortality for sea cucumbers with higher rates in juveniles has been described for *Isostichopus bandionotus* and *Holothuria* (*Microthele*) *fuscogilva*.^25^^,^^26^ Most estimates of instantaneous mortality for adult holothuroids range between 0.3 and 1.5 per year (reviewed in Hammond and Purcell^27^).

The most common method to estimate *M* in the wild is through mark–recapture studies. However, physical tags applied to holothuroids are only retained by the animals for a few weeks or months,^28^^,^^29^ rendering them ineffective over the long term. An alternative to physical tagging of individuals is to identify them by their natural pigment, spots, or body wall wrinkle patterns as applied to *Pearsonothuria graeffei*, *Bohadschia argus*, *Holothuria* (*M*.) *fuscogilva*, and *H. lessoni*.^27^^,^^30^^,^^31^^,^^32^^,^^33^

Twenty-four years ago, we proposed a technique to tag holothuroids based on genetic fingerprinting of individuals and re-sampling populations.^34^^,^^35^ We demonstrated that this technique can be used to derive growth rates for *H*. (*M*.) *whitmaei* which is harvested as “black teatfish”. After one year, we had high recapture rates of individuals from three sites on the Great Barrier Reef (GBR). Modeling suggested lifespans of several decades for this species (Uthicke et al.^35^), but that inference was uncertain given the short time between mark and recapture. Black teatfish is one of the highest-value (∼US$ 300 per kg dried^36^) sea cucumber species and has previously been overfished on the GBR^37^^,^^38^ and other regions, with some local extirpation noted.^39^ Populations of this species are also noted for the scarce observations of juveniles and the sensitivity to decreased spawner density (Allee effect), thereby indicating recruitment limitation.^37^^,^^38^ Consequently, *H*. (*M.) whitmaei* and two other teatfish species, which also appear to be recruitment limited and are overharvested globally, are listed on CITES Appendix II and classified as endangered or critically endangered on the IUCN Red list.^40^^,^^41^

We availed ourselves of the “biobanked” resource of frozen DNA extracts for the population of black teatfish tagged in 2000 and obtained a new set of samples from one site in 2024. Our aim was to determine if we could recapture and genetically identify the same individuals 24 years later as a means to assess the lifespan of this species. This is the first long-term application of genetic mark–recapture within the marine realm, representing an important advance to evaluate life histories in animals that cannot be physically tagged.

## Results

**Figure 1:**
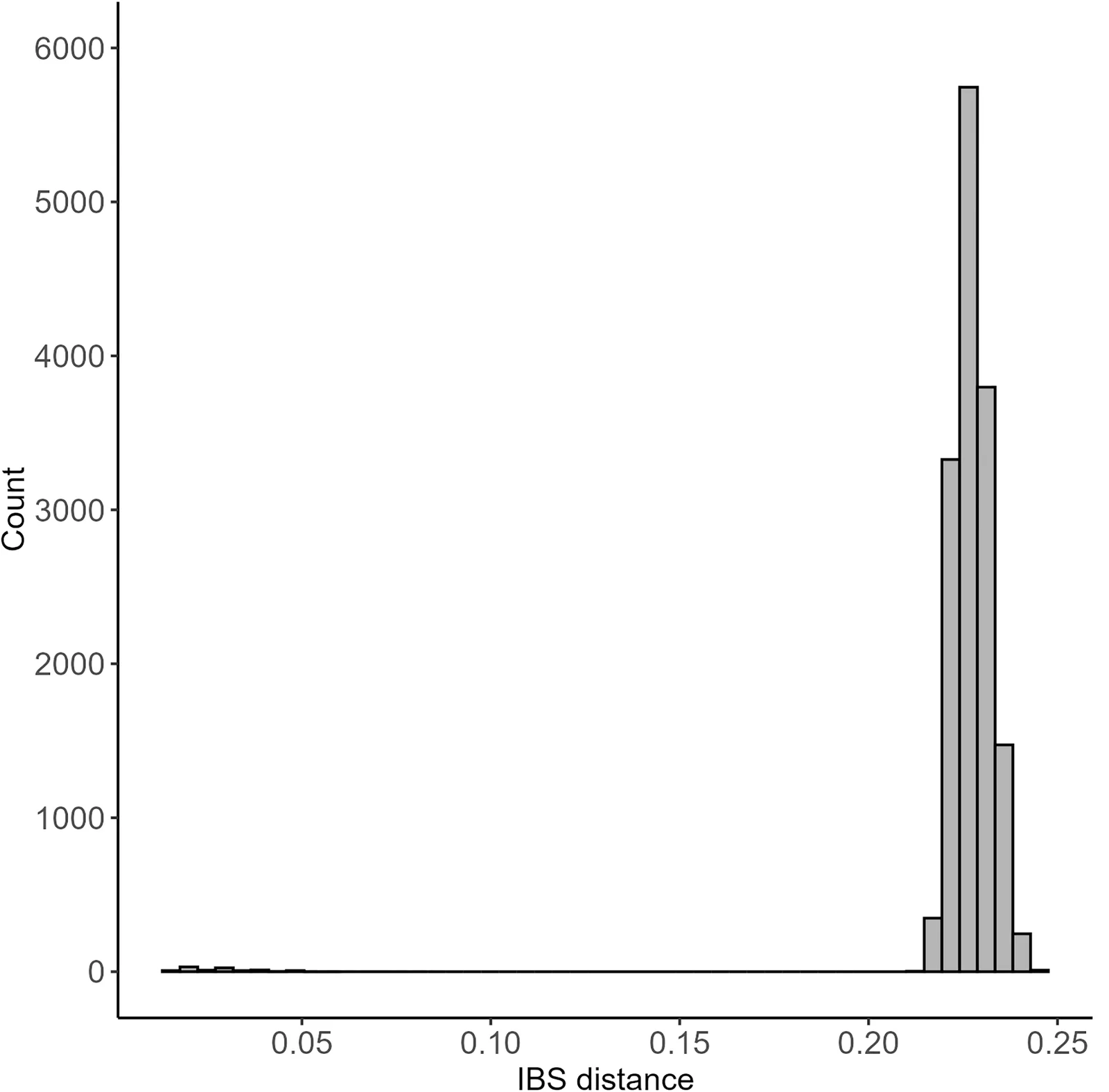
Pairwise isolation by state distances for all 15,051 comparisons in the SNP dataset for *H.* (*M.*) *whitmaei*

After filtering, 12,772 SNPs from 176 samples (including technical library repeats) were retained. The distribution of identity-by-state (IBS) differences between these profiles was distinctly bimodal, with the vast majority (14,949 out of 15,051, 99.3%) above 0.2 (Figure 1). A gap of distances exists to a smaller mode with values below 0.1. Thus, we chose values below 0.1 as a conservative threshold for identity.

The SNP profiles represent 56 samples (95% of samples assayed from that period) obtained in June 2000, 49 samples (100%) from December 2000, and 52 of the samples (98%) collected in September 2024.

**Figure 2:**
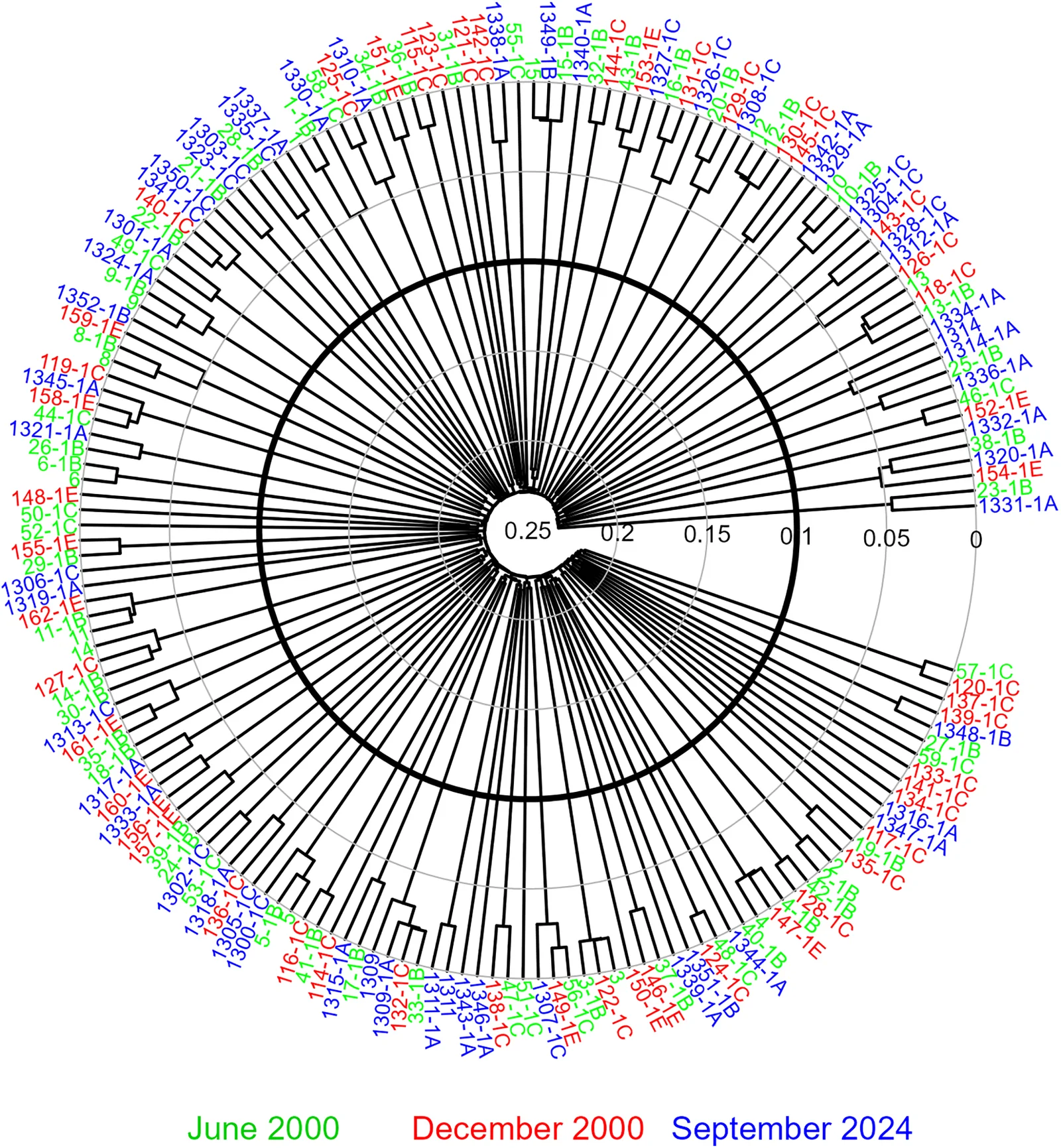
Circular plot of IBS distances between samples of *Holothuria whitmaei* from Lizard Island Numbers before the dash in the sample name are sample numbers; other numbers indicate repeated runs in different libraries. The label color indicates the three different sampling occasions. IBS distances smaller than 0.1 (indicated by the black circle) are considered the same individuals.

We obtained SNP profiles for 17 of the technical replicates (14 from 2000 and 3 from 2024) analyzed in two libraries. The IBS difference for each of these pairs was distinctly below 0.1 (Figure 2), confirming that IBS below that threshold corresponds to identity.

### Confirmation of previous matches

Out of the samples we analyzed, 23 were identified as the same individual between June and December 2000 (Figure 2 and Table S2) using the previous AFLP analysis. All matches were confirmed with the updated SNP method (Table S2). In addition, we identified six matches not previously identified (noting that not all samples amplified in the original AFLP data). Thus, 29 of 49 samples collected in December 2000 were also collected 6 months prior.

### Matches between new and historic samples

For the samples collected in 2024, 31 of the 52 individuals analyzed matched individuals from either (21) or both (10) collections in 2000 (Figure 2; Table S3). Thus, 60% of the samples collected in 2024 were confirmed to be at least 24 years old. The combined unique number of individuals collected in 2000 was 76. Estimates of total proportionate survival (*S*) using the comparisons to individual sampling occasions in 2000 or the pooled data were similar (Table 1) and around 0.4, with bootstrap confidence intervals ranging from 0.23 to 0.54.

**Table 1:** Initial and final sample size and number of genetic matches for comparison between the new (September 2024) samples of *Holothuria* (*Microthele*) *whitmaei* and those collected in June and December 2000 at Bird Islet, Lizard Island, and Great Barrier Reef

Parameter	2024 vs. June 2000	2024 vs. December 2000	2024 vs. all 2000
*N* initial (n1)	56	49	76
*N* final (n2)	52	52	52
Resamples (matches) (m2)	23	18	31
Time between samples (*a*, yr)	24.28	23.73	24.01
Total survival (*S*)	0.411 (0.286–0.536)	0.367 (0.225–0.510)	0.408 (0.302–0.513)
Annual instantaneous mortality (*M*)	0.037 (0.026–0.052)	0.042 (0.028–0.063)	0.037 (0.028–0.050)
Mean lifespan	27.3 (19.4–38.9)	23.7 (15.9–35.2)	26.8 (20.1–36.0)
p99 longevity	125.7 (89.3–179.1)	109.1 (73.1–162.3)	123.3 (92.5–165.7)

Survival over the total period, annual instantaneous mortality, mean lifespan, and the age where 1% of a cohort is still alive (p99 longevity) were calculated based on the proportion (*S*, m2/n1) resampled (genetic matches) at the second sampling occasion, following standard formulae for mark–recapture studies.^42^ Numbers in parentheses indicate the 95% confidence interval derived from 10,000 bootstrap replicates.

Assuming constant adult mortality, this survival indicated instantaneous mortality rates (*M*) between 0.037 and 0.042 per year (bootstrap values in Table 1), with a mean lifespan estimated between 24 and 27 years. This translates to a p99 longevity of 109–126 years (see Table 1 for confidence intervals). It should be highlighted that these parameter estimates are conservative because potential migration in and out the area and likely incomplete sampling would decrease survival (*S*) estimates and hence inflate the estimate of instantaneous mortality (*M*). This is supported by the fact that mean lifespan estimates of the population between 24 and 27 years appear low given we know from the recaptures that 60% of the animals were at least 24 years of age.

### Growth

**Figure 3:**
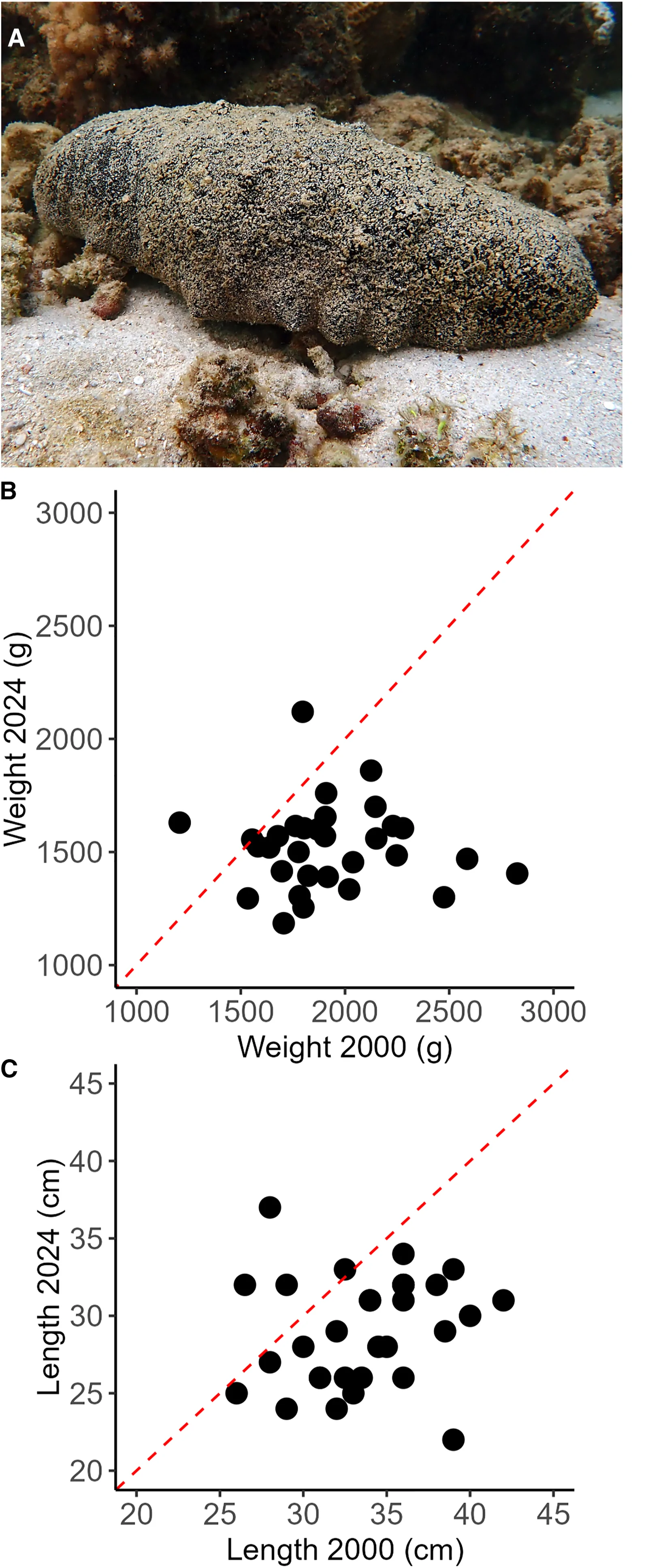
Differences in size of *H.* (*M.*) *whitmaei* between 2000 and 2024 (A) A photograph of *H.* (*M*.) *whitmaei* on Lizard Island (A, photo credit: S.W. Purcell) and changes in weight (B) and length (C) of individuals recaptured between 2000 and 2024. Values for 2000 were from individuals collected in either June or December, depending on which month they were found. We used average values in case the individual was previously found in both months. The red dotted line indicates the line of equal size or weight.

Linear model analysis indicated no significant relationship between both body weight (*F*_1, 29_ = 0.044, *p* = 0.8358, *R*^2^ < 0.001) or length (*F*_1, 29_ = 0.650, *p* = 0.4267, *R*^2^ = <0.001) of individuals collected in 2000 and 2024. Individuals were significantly lighter (on average 21%, *t* = −5.78, df = 30, *p* < 0.0001) and shorter (on average 14%, *t* = −5.12, df = 30, *p* < 0.0001) in 2024. Consequently, the weight and size ranges in 2024 were lower than those recorded in 2000 (Figures 3 and S1).

## Discussion

The aim of this study was to employ a genetic mark–recapture method using newly collected and archived samples to derive longevity estimates, mortality and growth rates for a high-value, critically endangered bêche-de-mer species to improve fishery management and conservation assessments of this and similar species. We found that a high proportion of the individuals sampled in 2024 were recaptures from 24 years earlier. Indeed, except for one study on clams analyzing two specimens recaptured ∼30 years after marks were notched on their shells,^43^ we could not find an invertebrate mark–recapture study of similar length. Although individuals of some sea urchin species can live to over 100 years,^12^ deriving at a similar conclusion for the black teatfish was surprising. This species is the first sea cucumber species known to surpass a longevity of a century, joining several other marine invertebrates exhibiting “centenarian” age.^2^ Based on growth data it was previously suggested that *H. whitmaei* reaches several decades of age,^35^ although subsequent fishery harvest models used much lower estimates.^44^^,^^45^ Our samples were collected from adults, many near the maximum size for the black teatfish, thus many may have been decades old at the time of the original sampling. At both time points in 2000 and 2024, no small individuals could be found, as is the case for other populations of *H*. (*M*.) *whitmaei*^37^ and other teatfish species.^31^ The lack of observed juveniles in populations of these species is an indicator for low recruitment rates.

Annual instantaneous mortality rates estimated here for *H*. (*M*.) *whitmaei* are very low compared to previous studies on sea cucumbers,^27^ and consequently the longevity estimate is high. The assumption of constant adult mortality is untested, although is used in most fishery modeling studies in invertebrates including sea cucumbers.^44^^,^^45^ Estimates or approximations of the natural instantaneous mortality rate are also used in fishery management and conservation planning for these species. Although the FAO Expert Panel for CITES recognized limited direct measurements of *M*, an approximation of 0.1 per year was suggested.^39^ Despite the earlier genetic fingerprinting mark–recapture studies indicating that natural mortality rates of black teatfish are “much lower than commonly assumed,” relatively high rates of 0.44 and 0.5 per year were subsequently applied for fishery assessments in teatfish species.^44^^,^^45^ For context, if *M* = 0.5, the population would be losing 39% of individuals each year, a rate equivalent to what we observed after all 24 years. Although an increase in mortality with age is possible, no examples of this are known for echinoderms. By contrast, some species of sea urchin even show negative senescence, i.e., reduced mortality at higher age.^23^ Our estimates for instantaneous mortality are likely inflated, because recapture rates will be reduced through potential migration of some individuals into and out of the sampling area and likely not capturing all individuals from the local population. This is also supported by the fact that recapture rates after 6 months in 2000 (29 out of 49 = 59%) were very similar to those after 24 years. Thus, we argue that the mortality of 0.04 is a maximum and the p99 longevity between 109 and 126 years are minimum estimates. This longevity may imply slow demographic turnover, suggesting that recovery of depleted populations would occur over multi-decadal timescales. In finfish stock assessments, compensatory population growth, e.g., through increased food for remaining adults leading to increased larval production and recruitment, is included in some models (e.g., Shepherd^46^). However, there is no known mechanism for this to occur in broadcast-spawning holothuroids. Recovery potential and time will depend on whether fishing was halted before populations reached critically low abundance to avoid Allee effects.^47^ This density is not known for *H.* (*M.*) *whitmaei*, because important biological parameters, such as fertilization kinetics, are under-studied in this species. However, based on surveys on reefs closed to fishing, it appears that densities of >12.5–20 ind.ha^−1^ are natural in suitable habitats.^37^^,^^48^^,^^49^

Surprisingly, most black teatfish specimens re-identified after 24 years did not grow but on average showed shrinkage in weight and length. Previous studies suggested that individual sea cucumbers can lose and regain weight from one season or year to another.^27^^,^^50^ This may be in response to circumstances specific to individuals or due to environmental conditions that favor or disfavor somatic growth. Environmental conditions for reduced size may include declining food conditions for these animals in the years before our recent sampling. Ocean heatwaves and cyclones have impacted our field site at Lizard Island in recent years, causing significant mortality of hard corals and reef degradation.^51^ While it is difficult to speculate on the potential flow-on effects of climate stress to deposit feeding macro-invertebrates, our data may imply that sea cucumbers may have been impacted somehow by the catastrophic loss of hard corals and overgrowth by algae. Asexual reproduction by transverse fission occurs in a minority of holothuroid species and can reduce the size of individuals.^8^ However, this reproductive mode does not occur in *H.* (*M.*) *whitmaei*^52^ and hence is unlikely to cause size and weight reductions observed here.

While low mortality rates and long lifespans in invertebrates are most noted for temperate and polar species, our study underscores that these life-history traits can be found in some tropical invertebrates. Indeed, lifespans of 50 years or more are also known for giant clams and coconut crabs of the tropical Pacific and Southeast Asia.^53^^,^^54^ Based on length-frequency analysis,^31^ a high longevity (57 years) was modelled for the closely related white teatfish *Holothuria* (*M*.) *fuscogilva*, which is also a high-value CITES-listed species. Our findings fill important knowledge gaps to inform the conservation of harvested tropical sea cucumbers and echinoderms in general. It appears that the physiological processes rendering a lack of senescence in sea urchins might also occur in other echinoderm classes.

### Conclusions

Estimates for the longevity and mortality of *H.* (*M.*) *whitmaei* are at least an order of magnitude higher and lower, respectively, than those reported for other sea cucumbers and which are used in population models produced to inform sustainable harvest. Although the black teatfish is on CITES Appendix II and is currently categorized as endangered on the IUCN Red List, some countries have continued harvesting and exporting this species. Continued fishing of black teatfish might only be sustainable if fisheries can curb harvests to a very small fraction (e.g., 1%–2%) of the virgin biomass annually. Our findings provide grounds for reassessment of conservation and management of many holothuroid species, which is especially urgent for the three teatfish species. The white teatfish was recently uplisted to Endangered on the IUCN Red List owing in part to new findings of a long generation length.^55^ Similarly, our findings underpin re-assessment of black teatfish. In addition, coral reefs suffer increasingly from climate change related pressures^18^ and individual stressors act cumulatively. Adding stress to the system by removing ecologically important species and the ecosystem services provided, such as by sea cucumbers,^15^ is incompatible with sustainable management of coral reefs.

### Limitations of the study

As mentioned earlier, one limitation of this study is that the sample location could not be sampled exhaustively, making it likely that some individuals collected in 2000 were not detected. In addition, data are only from one population.

## Resource availability

### Lead contact

Requests for further information and resources should be directed to and will be fulfilled by the lead contact, Sven Uthicke (suthicke@aims.gov.au).

### Materials availability

This study did not generate new unique reagents.

### Data and code availability

• Raw sequence data are available at the NCBI National Library of Medicine (Biosample accession numbers SAMN60877514–SAMN60877674).
• Initial sequence processing flowed standard procedures outlined in Parra-Salazar et al.^56^ with software provided at GitHub: https://github.com/NGSEP/NGSEPcore. The resulting genotype file (.vcr) and scripts to reproduce analyses and figures are available on the AIMS Data Repository: https://doi.org/10.25845/0FB7-BN12.
• Any additional information required to reanalyze the data reported in this paper are available from the lead contact upon request.

## Acknowledgments

We dedicate this publication to Drs. Anne Hoggett and Lyle Vail, former directors of the Lizard Island Research Station. Lyle and Anne have supported this study and many other studies by the authors through the decades with great enthusiasm. We thank the handling editor and two anonymous reviewers for their constructive comments, which helped to improve the manuscript.

## Author contributions

S.U. designed the experiment, conducted field work, bioinformatic analysis, and has written the first draft. M.G.C. conducted field work and laboratory analysis and contributed to the writing. S.W.P. and M.B. contributed to planning and design of the experiment, writing, and interpretation of the data.

## Declaration of interests

The authors declare no competing interests.

## STAR★Methods

### Key resources table

**Table:** 

REAGENT or RESOURCE	SOURCE	IDENTIFIER
**Biological samples**		

New samples (from 2024) 53 skin samples of *Holothuria (Microthele) whitmaei*. In ethanol	Bird Islet, stored at AIMS	LG1300-1352
Original sample (from 2000): 108 skin samples of *Holothuria (Microthele) whitmaei*. Frozen (−80°C)	Bird Islet, stored at AIMS	LG1-59, LG114-162

**Deposited data**		

Raw sequence data	NCBI National Library of Medicine	Biosample accession numbers SAMN60877514- SAMN60877674
Vcf file	AIMS Data Repository	DOI: https://doi.org/10.25845/0FB7-BN12

**Software and algorithms**		

Raw sequence processing	Initial sequence processing flowed standard procedures outlined in^56^ with software provided at gitgub	https://github.com/NGSEP/NGSEPcore
R scripts for figures and analysis	AIMS Data Repository	DOI: https://doi.org/10.25845/0FB7-BN12

### Experimental model and study participant details

The present study is conducted at Bird Islet, one on the original sample sites from 2000.^34^^,^^35^ DNA from the 59 *Holothuria* (*Microthele*) *whitmaei* samples collected at that site in June 2000 were stably stored at −80°C and available for reanalysis (LG 1–59). In addition, DNA was available from 49 (out of original 59) samples collected in December 2000 (LG 114–162). The individuals found at both sampling occasions in 2000 were all large adults (26–42 cm in length, Figure S1) near the maximum size for this species (Wolfe and Byrne 2022). Whereas Conand^57^ found that small animals lost their cream blotches and became all black around the size of sexual maturity, all the individuals sampled in the present study were solid black. We collected tissue samples from 53 *H.* (*M.*) *whitmaei* from this same location in September 2024 (LG 1300–1352) (collected on 16/09/2024). The individuals found in 2024 were also exclusively large adults (22–37 cm in length, Figure S1). The center of the previous search location (based on the GPS position, notebook sketches and site knowledge) was marked with a buoy. Three snorkelers collected animals within approximately 100 m of the buoy. As in the original study, three small (1–2 cm in length, 0.5 cm wide) and shallow skin samples from the ventral body wall were collected and kept in 100% ethanol, changed once after 24 h. Individuals were weighed using a Pryml digital Scale (accurate to 5 g), length measured with a tape ruler and carefully returned to the seafloor within 10 m of the buoy marking the center of the search area. Sample collection in 2024 was conducted under GBRMPA permit G23/47045.1.

### Method details

DNA of a small subsample (5–15 mg) of epidermis followed the protocol used for the historic samples,^34^ which uses Qiagen DNeasy extraction kits with the standard protocol, but using twice the recommended amount of proteinase K (40 μL at a concentration of 20 mg mL^−1^). In addition, extracts were cleaned using CL 6 B Sepharose spin columns. A final purification step was carried out using a 1 : 1 ratio of SPRIselect Beads (Beckman Coulter, USA) to remove smaller degraded fragments and concentrate samples.

Final cleanup, library preparation and sequencing was done at the Australian Genome Research Facility (AGRF). A ddRAD based library preparation protocol^58^ was used for processing. Briefly, DNA digestions used EcoRI and HpyCH4IV enzymes chosen after an initial trial of several enzyme combinations. This was followed by ligation of barcoded adapters compatible with restriction sites overhang, and size selection of pooled digested-ligated fragments and amplification of the library via PCR using indexed primers.

Sequencing was performed on 10 B 300 cycle lanes on the Illumina NovaSeq X platform, yielding 150 bp paired end reads.

Initial bioinformatics was conducted at AGRF using the DeNovoGBS function from NGSEP Version 4.2.0 (07/01/2022)^56^ resulting in a vcf file with 224,347 SNPs.

For relatedness analysis, we removed all samples with less than 2 M reads and missingness of variable sites of 60%. We reduced the number of SNPs further by setting minimum allele frequencies required to 0.05 and only selected SNPs present in at least 97% of samples.

A total of 220 samples or repeats were sequences across 5 libraries in August (4 libraries) and November 2025 (Table S1). Preliminary analysis indicated samples LG6 and 7 within the June 2000 collection had the same genotype. Re-inspection of original AFLP patterns confirmed this match. The same genotype would generally indicate a clone. Although asexual reproduction by transverse fission is known in a small number of holothuroids, it has never been reported for *H. whitmaei*.^8^ The thick and rigid body wall and large body size makes this species an unlikely candidate for transverse fission. Given the two samples had subsequent sample numbers (LG6 and 7), we suggest the reason for the same multi-locus genotype is based on an error in sampling and fixation and removed LG7 from further analysis.

Pairwise identity-by-state (IBS) was calculated as the mean proportion of shared alleles across loci, coding genotypes (g) as alternate-allele counts (0, 1 or 2). Thus, per-locus, IBS was computed as

$$\text{IBS}=\left(2-\left|g_{i}\;–\;g_{j}\right|\right)/2$$

and genome-wide IBS was computed as average of the per locus genotypes.^59^ IBS distance was calculated as 1-IBS. Information on the number of animals initially collected and ultimately included in the analysis after quality control is given in Figure S2. Original sample number and the number of reads obtained are given in Data Supplement 1.

### Quantification and statistical analysis

Although mortality rates in echinoderms may change over time, most fisheries assessments or publications use a constant mortality, or assume higher rates in juveniles followed by constant rates in adults.^25^^,^^26^ Given that all the individuals sampled in 2000 were large adults, well beyond the juvenile stage, we only estimate a constant rate of mortality for adults here, based on the proportion of individuals we re-detected after 24 years.

We estimated mortality and longevity for three time periods. Two of these compare matches between September 2024 with June and December 2000 separately. We then combined samples from the two sampling occasions in 2000 and derived the total number of unique matches at the second sample (m2). In this instance, the initial sample size (n1) was the number of samples successfully genotyped with the SNP-method. We estimated survival (*S*) using the proportion of initially sampled individuals recaptured (m2/n1) as proposed in most standard models e.g.,.^42^ Although the sampling effort was roughly similar compared to individual historic sampling occasions, it is likely that we did not exhaustively sample all individuals from the population in 2024. Thus, estimates derived for *S* are likely conservatively low, and thus estimates for *M* (annual instantaneous mortality) are maximum estimates since these two are inversely related.

Based on survival, annual instantaneous mortality (*M*) was calculated as

M=−ln(S)/a

with *a* = 24.48 or 23.73 years between studies for June and December 2000, respectively. For the analysis pooling all 2000 samples we assumed the average of these values (*a* = 24.01). We then calculate the mean lifespan as 1/*M*. We estimated the age where 1% of a cohort still alive (p99 longevity) as:

−ln(0.01)/M.

95% bootstrap confidence intervals were obtained from the 2.5th and 97.5th percentiles of 10,000 non-parametric bootstrap resamples of *S* using the boot library in R.^60^

To compare weight and length of individuals re-identified we used a general linear model of size in 2020 vs. size in 2000. Because graphic inspection suggested most individual were reduced in size in 2024 we used a one-tailed paired *t* test to test whether weight and length were significantly reduced in 2024. Analysis and figures were prepared in R.^61^

## Supplemental information

Document S1. Figures S1 and S2 and Tables S2 and S3

Table S1. List of samples and repeats analyzed
