# Supplementary material for: Extreme longevity revealed by 24-year genetic mark–recapture challenges conservation of endangered sea cucumbers
Source: iScience. 2026 Sep 15;29(10):117505. doi: 10.1016/j.isci.2026.117505 (PMC13594949; doi:10.1016/j.isci.2026.117505)
Supplement: Supplementary file 1 [file mmc1.pdf]

iScience, Volume *29*

Supplemental information

Extreme longevity revealed by 24-year genetic mark–recapture challenges conservation of endangered sea cucumbers

Sven Uthicke, Maria Gomez Cabrera, Maria Byrne, and Steven W. Purcell

Supplementary Material: Uthicke et al:

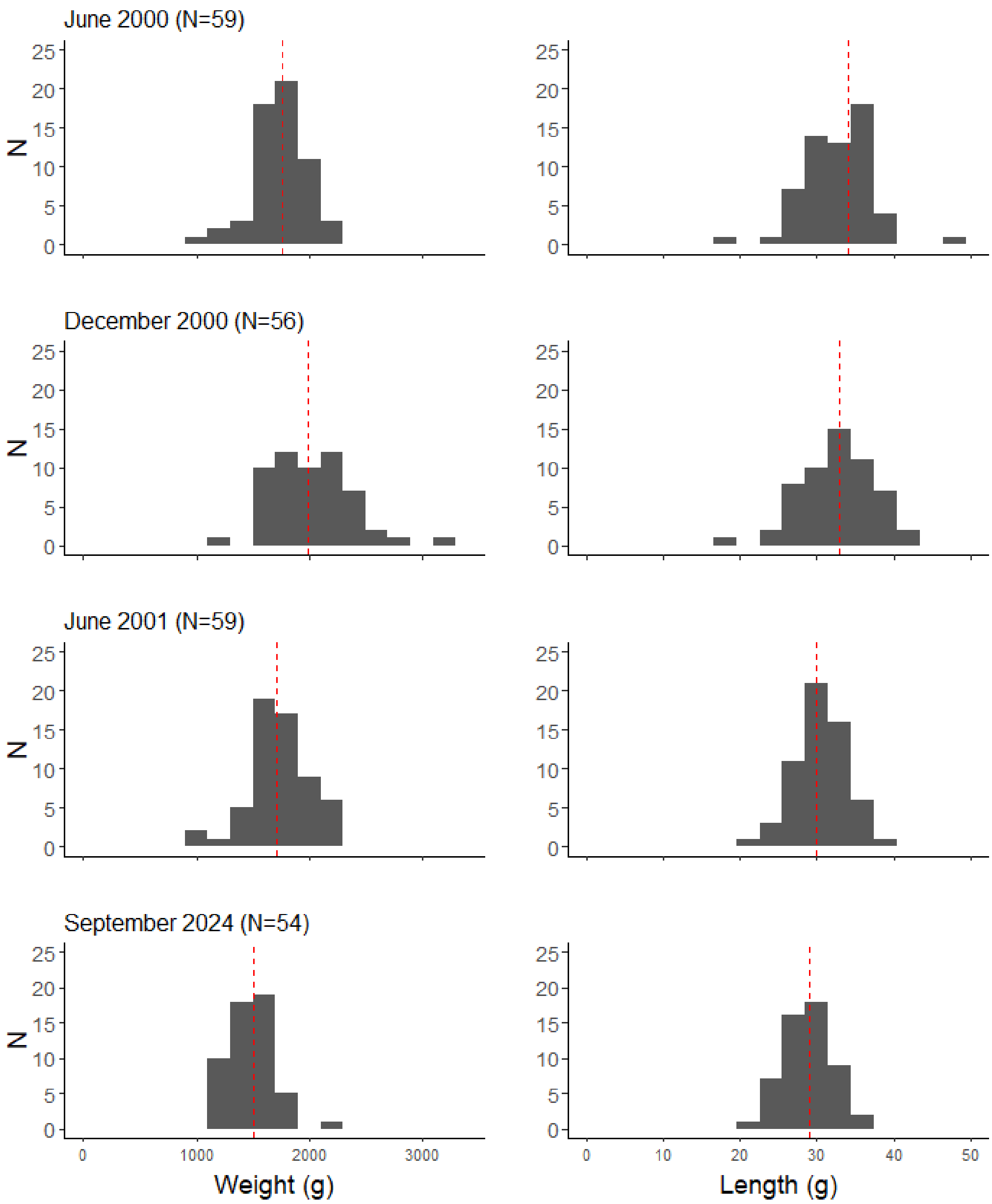

Supplemental Figure S1. Weight and length of *Holothuria (Microthele) whitmae*i. Size frequency distribution al all individuals of *Holothuria (Microthele) whitmaei* captured during the original three sampling occasions in 2000 and 2001, and in the newly collected samples. The red dashed vertical line indicates the median weight or length.

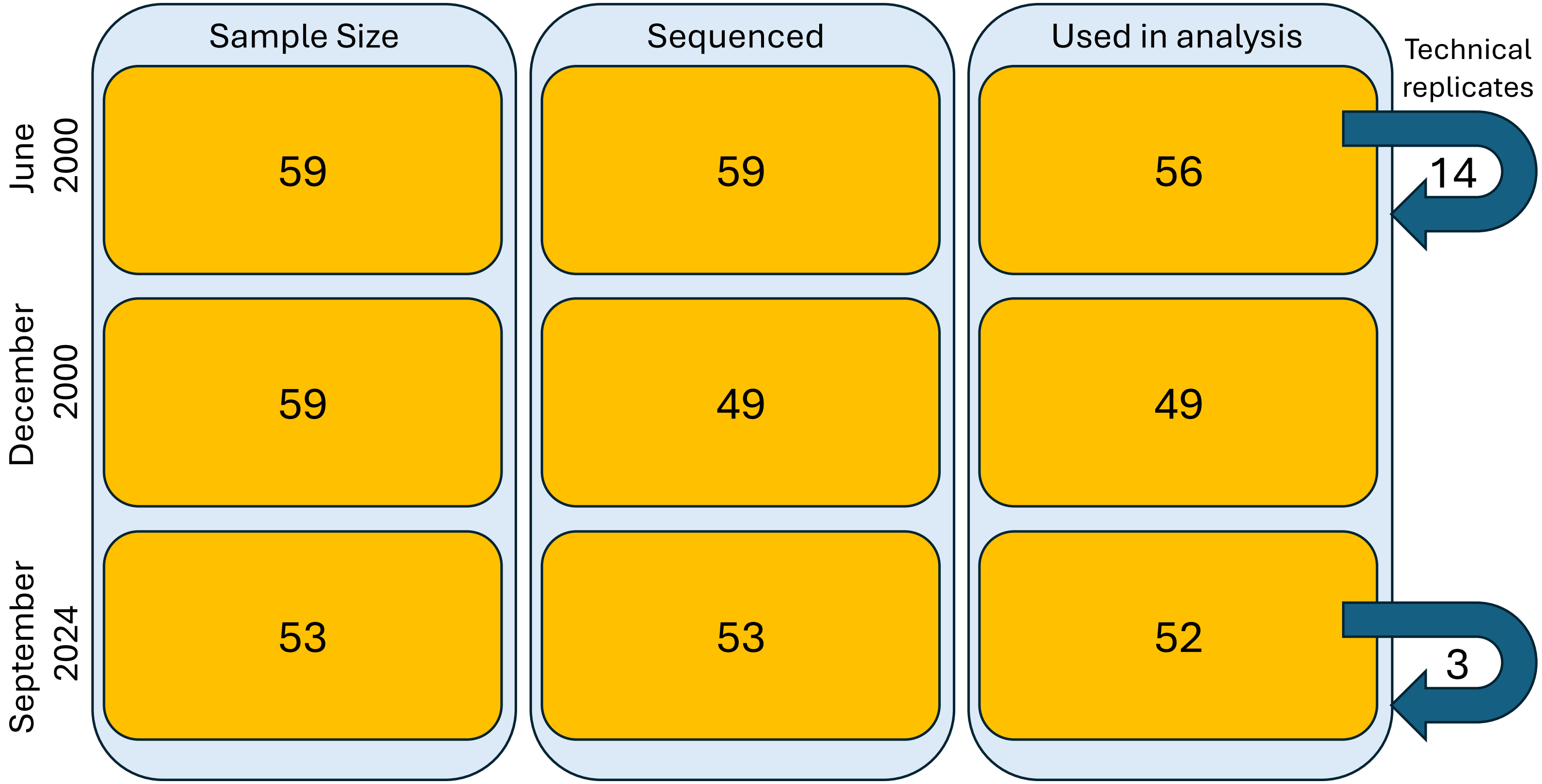
Sample Size
Sequenced
Used in analysis
Technical replicates
June 2000
December 2000
September 2024
59
59
56
14
59
49
49
53
53
52
3

Supplemental Figure S2 Flow chart of sample collection and analysis. Diagram depicting the number of samples collected in the field, the number sequenced and the number analysed (e.g., good quality SNPs obtained and included in analysis at the three sample occasions. Technical replicates were run on separate libraries.

Supplemental Table S2 Historic matches of *Holothuria (Microthele) whitmaei* individuals. Comparison of matches between June 2000 and December 2000 using the Amplified Fragment Length Polymorphism (AFLP) method previously published, and the current SNP method. Numbers in the June and December column are original sample identifications.

June 2000	December 2000	AFLP match	SNP match
3	122	+	+
4	147	+	+
8	159	-	+
12	130	+	+
13	118	-	+
14	127	+	+
16	131	+	+
19	135	+	+
20	129	+	+
22	140	+	+
29	155	-	+
32	144	+	+
33	132	+	+
34	151	+	+
35	161	+	+
36	115	+	+
37	146	+	+
38	154	-	+
39	157	-	+
41	116	+	+
42	128	+	+
43	153	+	+
44	158	+	+
46	152	-	+
47	138	+	+
48	124	+	+
56	149	+	+
57	120	+	+
58	125	+	+

Supplemental Table S3 New matches of *Holothuria (Microthele) whitmaei*. Matches of recent samples with historical samples, for IBS distances see Fig 2. In total 23 samples matched specimens collected in June 2000 and 18 those collected in December 2000.

September 2024	June 2000	December 2000
1300	5	
1301	49	
1302	53	
1307	56	149
1308	20	129
1309	33	132
1310	58	125
1312		126
1313	35	161
1315	17	
1317		160
1318		136
1320	38	154
1321	26	
1323	21	
1324	9	
1325	10	
1326	16	131
1327	43	153
1328		143
1330	1	
1331	23	
1333		156
1335	28	
1336	25	
1338		142
1341	22	140
1342		145
1345	44	158
1348		139
1349	15	
